# Risk factors and risk-stratification model of biochemical failure in patients with positive surgical margin after robot-assisted radical prostatectomy

**DOI:** 10.1007/s11701-025-02426-x

**Published:** 2025-06-04

**Authors:** Yuta Yamada, Yuji Hakozaki, Naohiro Makise, Jimpei Miyakawa, Yoichi Fujii, Naoki Kimura, Kazuma Sugimoto, Jun Kaneko, Kazuki Maki, Taketo Kawai, Shigenori Kakutani, Aya Niimi, Jun Kamei, Satoru Taguchi, Daisuke Yamada, Haruki Kume

**Affiliations:** 1 Department of Urology, Graduate School of Medicine, The University of Tokyo, 7-3-1, Hongo, Bunkyo-Ku, Tokyo 113-8655 Japan; 2 Department of Urology, Chiba Tokushukai Hospital, Funabashi-Shi, Chiba, Japan; 3 Division of Surgical Pathology, Chiba Cancer Center, Chiba-Shi, Chiba, Japan; 4 Department of Urology, International University of Health and Welfare Ichikawa Hospital, Ichikawa-Shi, Chiba, Japan

**Keywords:** Robot-assisted radical prostatectomy, Positive surgical margin, Biochemical recurrence, Risk factors

## Abstract

The purpose of this study was investigating the risk factors of biochemical failure in cases with positive surgical margins after robot-assisted radical prostatectomy (RARP). We identified 630 cases of RARP that had been performed in a single institution from November 2011 to December 2017. A total of 145 cases showed positive surgical margin after RARP. After excluding patients that had undergone adjuvant therapies (androgen deprivation or radiation), 114 cases were evaluated. Clinical and pathological parameters were investigated in this cohort and were analyzed to identify factors predicting biochemical failure. Subsequently, we developed a risk classification based on these risk factors. The median values of PSA, age, preoperative prostate volume, were 67 (internal quartile range [IQR]: 65–72) years, 9.3 (6.5–12.0) ng/mL, and 37 (25–45) cm^3^, respectively. There were 53 and 61 patients with pT2 and pT3, respectively. The International Society of Urological Pathology grade group at surgical margin (SM) ≥ 5 (hazard ratio [HR] 3.37, 95% confidence interval [CI] 1.48–7.68), and max length of cancer at SM ≥ 4 mm (HR 2.59, 95% CI 1.27–5.32), and nadir PSA ≥ 0.03 (HR 8.66, 95% CI 4.66–16.1) were identified as factors predicting biochemical failure. Subsequently, a risk-stratification model was developed using these factors. The model showed a C index of 0.801. This study identified risk factors predicting biochemical failure in patients with positive surgical margins after RARP.

**Supplementary Information:**

The online version contains supplementary material available at 10.1007/s11701-025-02426-x.

## Introduction

Radical prostatectomy has shifted towards a robot-assisted approach in the last decade [1]. Compared with the open surgical approach, robotic surgery provides better magnification and manipulation in small and narrow spaces that may lead to favorable oncologic and functional outcomes [2]. Conventionally, the Cancer of the Prostate Risk Assessment (CAPRA) score is a good diagnostic tool to predict prostate cancer recurrence based on the results of radical prostatectomy [3]. However, this model is based on a cohort that includes patients undergoing open radical prostatectomy. Therefore, a new risk model based on robot-assisted radical prostatectomy (RARP) is needed.

Recently, we reported reduced positive surgical margin (PSM) rates in pT2 cases subjected to the RARP procedure [2]. Since PSMs are expected to be related to an elevated risk of distant metastatic recurrence [4] and biochemical recurrence [5] after radical prostatectomy, the priority of this surgery would be the complete resection of the prostate. Nevertheless, up to 10–15% of these patients harbor PSM [5].

The condition of the surgical margin (SM), whether positive or negative, is crucial when deciding on additional treatment and follow-up because the background of biochemical recurrence differs between the two situations. In patients with a negative SM after RARP, micro-metastasis is more likely to be the cause of biochemical recurrence. Indeed, we previously reported the risk factors of biochemical failure (BCF), developed a risk-stratification model, and created a nomogram [6]. Conversely, it is more complicated in patients with PSMs. It is imperative that we consider both surgery-related and host-related factors in this specific spectrum of patients. For instance, a case may involve advanced cancer, suboptimal surgical quality, or both.

Given this, we were motivated to investigate the factors that predict BCF in this specific spectrum of patients. Although there are previous reports regarding this topic, some reports were from the open radical prostatectomy (ORP) era, and some did not assess the findings of the SM-site and develop risk models. Factors associated with PSM may be different from the ORP-era where PSM were scattered throughout ubiquitous parts of the prostate [7]. Notably, in the ORP-era, PSM were polarized to either the apex or the basal area of the prostate which led to a significant decrease at the lateral part of the prostate [7]. We also postulated that indications of cauterization at the site of the surgical margins may also influence the status of BCF. Taken together, the objective of the present study was to determine the risk factors associated with BCF in patients with PSM.

## Materials and methods

### Patient characteristics

**Fig. 1:**
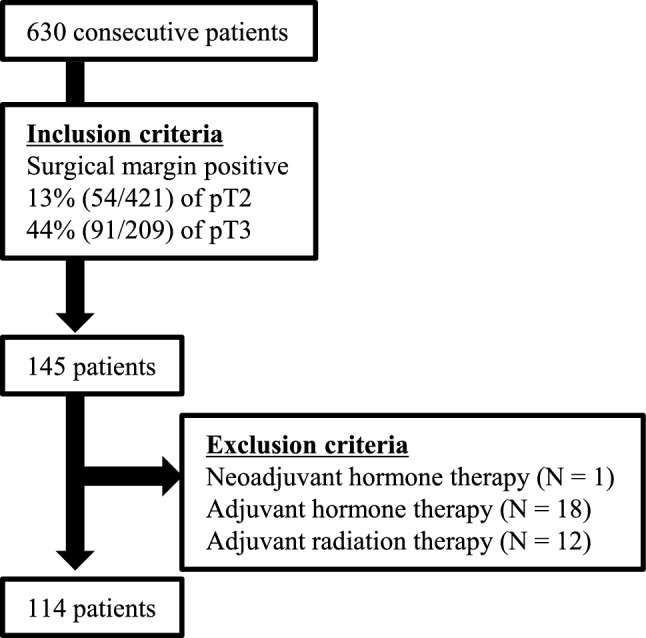
Flowchart of the inclusion and exclusion criteria

RARP was performed on 630 consecutive patients at a single institution from November 2011 to December 2017. A total of 145 cases showed PSM after RARP. After excluding patients who had undergone either neoadjuvant or adjuvant therapies (androgen deprivation or radiation), a total of 114 cases were included in the study (Fig. 1). The medical records of these patients were retrospectively investigated and the clinical data were prospectively collected and added to our cohort that had been currently used in our previous literature [8]. BCF was defined as two consecutive PSA values elevated above 0.2 ng/mL. The staging of prostate cancer was determined according to the American Joint Committee on Cancer (AJCC) TNM staging system [9]. All the subjects were followed at our outpatient clinic for at least 6 months. “Indication of cauterization at the site of PSM” is a diathermy artifact caused by extensive cauterization at PSM, which was defined as “elongation and hyperchromasia of nuclei of epithelial cells, tissue fragmentation, and/or loss of nuclear and cytoplasmic detail” [10].

All subjects signed a written consent form allowing use of their medical records for research. The present study was approved by the institutional review board (Ethics Committee of the Tokyo University Hospital)(#3124). Clinical data were anonymized after collecting from the medical records. This study follows the Helsinki Declaration.

### Surgical procedure of RARP

The da Vinci surgical robot system (Intuitive Surgical Incorporation, Sunnyvale, CA) was used for surgery. Transperitoneal RARP was done as previously reported [11, 12]. After dissection of the peritoneum at the center of the abdomen, the bladder neck was dissected from the center. The isolation of the seminal vesicles and vas deferens followed. The dorsal vein complex (DVC) was transected with monopolar scissors and occasionally point-coagulated when arterial bleeding occurred. The DVC was then managed by a vertical running suture with a 3–0 absorbable monofilament. Neurovascular bundle preservation was determined after a discussion between the surgeons and the patient. The maximum length of the urethra was preserved. We did not use an anterior stitch in any of the cases. Rocco’s stitch was used for reinforcing the posterior pelvic floor [13]. Urethro-vesical anastomosis was performed using a 3–0 absorbable monofilament. A total of 14 surgeons performed RARP in the present cohort. Surgeon volume was defined as the number of RARP procedures performed per surgeon.

### Statistical analyses

Statistical analysis was performed using R statistical software version 4.3.2. Univariate and multivariate Cox proportional hazards regression models were assessed to find independent risk factors predicting prostate-specific antigen (PSA) recurrence-free survival. Kaplan–Meier curves of the PSA recurrence-free survival were drawn and analyzed with a log-rank test. A *P* value of < 0.05 was defined as statistically significant. A nomogram for predicting 3-year recurrence free survival was created, and a calibration plot was constructed using the *rms* package.

## Results

The clinical demographics of 114 patients with PSM after RARP are shown in Table 1. The median follow-up duration was 59 months (interquartile range [IQR]: 18–66). The median values of age, preoperative prostate volume, and preoperative PSA were as follows; age: 67 (IQR: 65–72) years, preoperative prostate volume: 37 (25–45) cm^3^, and preoperative PSA: 9.3 (6.5–12.0) ng/mL, respectively. There were 53 patients with pathological stage T2 (pT2) and 61 patients with pT3.

**Table 1:** Baseline characteristics (*N* = 114)

Variable	Median value (interquartile range) or number of cases (%)
Follow-up (months)	59 (18–66)
Age (year)	67 (65–72)
Preop- PSA (ng/ml)	9.3 (6.5–12.0)
Prostate volume (g)	37 (25–45)
Preop- ISUP score	
1–3	83 (72.8)
4	10 (8.8)
5	21 (18.4)
Clinical T stage	
cT1	87 (76.3)
cT2	23 (20.2)
cT3a	4 (3.5)
Pathological T stage	
pT2	53 (46.5)
pT3a	57 (50.0)
pT3b	4 (3.5)
Perineural invasion	93 (81.6)
Seminal vesicles invasion	4 (3.5)
Lymphovascular invasion	62 (54.4)
Maximum diameter of tumor (mm)	21 (17–28)
ISUP score at the site of PSM	
1–3	69 (60.5)
4	31 (27.2)
5	14 (12.3)
Maximum length of cancer at surgical margin (mm)	4 (2–6)
Multifocal positive margin	60 (52.6)
Indication of cauterization at the site of PSM	59 (51.8)
Surgeon volume	25 (12–48)
Localization of positive margin	
Apex	62 (54.4)
Base	35 (30.7)
Lateral	27 (23.7)
Pelvic lymph node dissection	29 (25.4)
Lymph node metastasis	0 (0.0)

*Preop* preoperative, *PSA* prostate specific antigen, *ISUP* International Society of Urological Pathology, *PSM* positive surgical margin

Table 2 shows the univariate and multivariate analyses for identifying the risk factors associated with BCF after RARP. “Pathological T stage (≥ 3)”, “ISUP grade group of the entire tumor”, “ISUP grade group at SM (≥ 5)”, “Maximum length of cancer at SM (≥ 4 mm)”, “Nadir level of PSA after surgery (nadir-PSA)”, and “Indication of cauterization at SM” were identified as significant factors in the univariate analysis. Multivariate analysis revealed that “ISUP grade group at SM ≥ 5” (HR 3.37, 95% CI 1.48–7.68), “Maximum length of cancer at SM ≥ 4 mm” (HR 2.59, 95% CI 1.27–5.32), and nadir-PSA ≥ 0.03 (HR 8.66, 95% CI 4.66–16.1) remained significant factors that predicted BCF.

**Table 2:** Univariate and multivariate analyses of prognostic factors of PSA recurrence-free survival

		Univariate			Multivariate		
Variable		Hazard ratio	95% index	*P* value	Hazard ratio	95% index	*P* value
Age, years	≥ 70 vs. < 70	0.65	0.32–1.30	0.223			
PSA, ng/mL	≥ 10 vs. < 10	0.73	0.40–1.35	0.319			
Pathological T stage	≥ pT3 vs. ≤ pT2	2.38	1.28–4.42	0.006*	1.56	0.80–3.05	0.193
Perineural invasion	Positive vs. Negative	2.22	0.88–5.61	0.093			
Seminal vesicle invasion	Positive vs. Negative	1.93	0.47–8.01	0.364			
Lymphovascular invasion	Positive vs. Negative	1.41	0.78–2.53	0.255			
Pathological ISUP grading group of the entire tumor	≥ 4 vs. < 4	2.68	1.47–4.87	0.001*			
	≥ 5 vs. < 5	2.28	1.17–4.43	0.015*			
MTD, mm	≥ 20 vs. < 20	1.16	0.64–2.11	0.625			
MTD-mGS, mm	≥ 20 vs. < 20	1.14	0.63–2.04	0.666			
ISUP grading group at SM	≥ 3 vs. < 3	1.25	0.62–2.53	0.527			
	≥ 4 vs. < 4	1.76	0.97–3.21	0.064			
	≥ 5 vs. < 5	3.32	1.58–6.97	0.002*	3.37	1.48–7.68	0.004*
Max length of cancer at SM, mm	≥ 4 vs. < 4	3.77	1.91–7.43	< 0.001*	2.59	1.27–5.32	0.009*
Nadir PSA, ng/mL	≥ 0.02 vs. < 0.02	6.41	3.45–11.9	< 0.001*			
	≥ 0.03 vs. < 0.03	7.43	4.09–13.5	< 0.001*	8.66	4.66–16.1	< 0.001*
Indication of cauterization at the site of PSM	Positive vs. Negative	2.00	1.11–3.62	0.022*	1.79	0.96–3.32	0.067
Surgical volume	≥ 30 vs. < 30	0.60	0.33–1.10	0.100			

*PSA* prostate specific antigen, *ISUP* International Society of Urological Pathology, *MTD* maximum tumor diameter, *mGS* maximum Gleason’s score, *SM* surgical margin

**Fig. 2:**
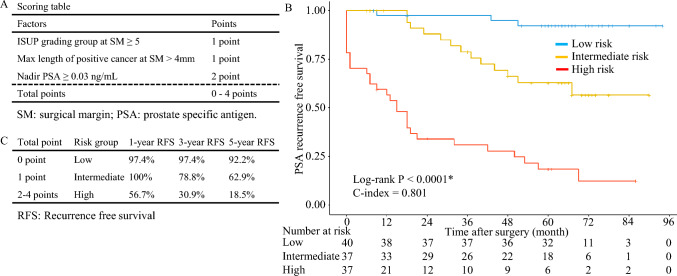
Kaplan–Meier curves according to the risk stratification **A** Scoring table for each risk factor **B** Kaplan–Meier plot of prostate-specific antigen (PSA) recurrence-free survival according to the risk groups **C** PSA recurrence-free survival based on the risk groups

We developed a risk model according to the coefficients of the factors (ISUP grade group at SM ≥ 5, maximum length of cancer at SM ≥ 4 mm, and nadir-PSA ≥ 0.03). One point was given to the ISUP grade group at SM ≥ 5 or maximum length of cancer at SM ≥ 4 mm and 2 points were given to nadir PSA ≥ 0.03. “Low risk” was defined as 0 points, “intermediate risk” as 1 point, and “high risk” as 2–4 points (Fig. 2A). Based on this categorization, Kaplan -Meier estimates were calculated. Estimates of BCF-free survival at 12, 36, and 60 months for the low-, intermediate-, and high- risk groups were as follows: low risk: 97.4%, 97.4%, and 92.2%; intermediate risk: 100%, 78.8%, and 62.9%; and high risk: 56.7%, 30.9%, and 18.5%, respectively (Fig. 2A, C, Supplementary Fig. 1). Our risk model showed the C index of 0.801. Based on the identified risk factors, a nomogram and calibration plot were constructed for predicting 3-year BCF free survival (Supplementary Fig. 2 A and 2B).

## Discussion

PSA recurrence occurs in two main ways. In patients with negative SM, there is a very low possibility that the local region of the prostate may be responsible for the recurrence since there is no remnant cancer. In this spectrum of patients, micro-metastasis may be the main reason for PSA recurrence. However, in cases with PSM, there is a high probability of remnant cancer cells at the PSM site, which makes us to care about both elements regarding PSA recurrence.

The present study showed that the ISUP grading group at SM, maximum length of cancer at SM, and nadir PSA were the significant factors associated with BCF in RARP patients with PSMs. These two factors reflect the burden and biological aggressiveness of residual cancer at the PSM. The latter factor, nadir-PSA, also includes the effect of micro-metastasis. We further developed a risk model that predicts BCF. This model may support decision-making on indication of adjuvant therapies.

In the current National Comprehensive Cancer Network (NCCN) guideline [14], PSM is an indication of adjuvant hormone/radiation therapy. However, hormone therapies may cause adverse events such as hot flushes [15], fatigue [15], overweight [15], cardiovascular disease [16], osteoporosis [17], sarcopenia [18], and mental disorders [19]. Radiation therapy may also cause higher damage to the pelvic organs after resection of the prostate compared to when the prostate is not resected. We have previously reported a higher rate of radiation-cystitis in cases with radiation therapy after radical prostatectomy (RP) when compared with initial radiation therapy [20]. We concluded that radiation after prostatectomy leads to more exposure to adjacent normal tissue of the bladder and rectum which elevates the risk of radiation cystitis. Therefore, it would be clinically significant to optimize adjuvant therapies in patients showing PSM after RP. In the present study, 48 out of 115 (42%) men were categorized as a low-risk group. Given that PSA recurrence-free survival was very high in the low-risk group, patients belonging to this group may harbor fewer benefits from adjuvant therapies. On the contrary, the 5-year recurrence-free survival rate was only 18.5% in the high-risk group, so these patients may benefit from early adjuvant therapies. Future studies are required to evaluate this hypothesis.

The present study is unique from previous studies since, the risk model also includes nadir-PSA as one of the factors. Nadir-PSA may reflect on the remnant cancer cells either in the form of micro-metastasis or remnant cancer cells at the PSM site, which should also be accounted for in cases with biochemical recurrence. The present study was in line with the report by Su et al. showing that nadir-PSA was a significant factor as well as platelet/lymphocyte ratio and pathological grade [21].

It is natural to find that the “maximum length of PSM” is one of the significant factors predicting BCF in cases with PSM. The longer length shows that there is a greater number of remnant cancer cells expected at the PSM site. This factor was reported as a significant BCF risk in previous studies [22, 23]. Shikanov et al. showed the length of PSM as a significant factor of BCF in patients undergoing RARP [22]. Another study confirmed this by reporting that non-focal PSM, defined as length of PSM over 1 mm, was significantly associated with BCF in patients undergoing RARP [23].

Another factor relevant to the BCF is the “ISUP grade group” of the PSM. Preisser et al. found that Gleason ≥ 4 at the SM and length of PSM were independent predictors of biochemical recurrence after RP [24]. A meta-analysis including 10 studies also showed that Gleason grade 4 at PSM was significantly associated with biochemical recurrence compared with Gleason grade 3 [25]. In the present study, the higher grade group (≥ 4 or ≥ 5) showed higher risk of biochemical recurrence.

This study has some limitations. This study is retrospectively investigated and it also has small numbers of cases. A larger external validation study may therefore be required to confirm this risk-model in the future. Secondly, the characteristics of studies involved with surgery always have heterogeneity among institutions since the methods of surgery differ among them. However, our approach of surgery is in line with the popular method of RARP and may be recommended in other institutions for the evaluation of the model. Another limitation is the accuracy required to detect metastasis at the time of surgery. Although PSMA-PET is a strong diagnostic tool for detecting metastasis, it was not performed in this cohort, due to the insurance coverage in Japan. Pelvic lymph node dissection (PLND) is the most powerful tool for detecting pelvic lymph node metastasis and PLND was performed in the indicated cases in the cohort. Distant metastasis was detected by CT, but may have a weaker power to detect metastasis than PSMA-PET.

In conclusion, we identified risk factors predicting BCF in a group of PSM patients and developed a risk-stratification model. Our risk model shows that both the status of margin (size and aggressiveness of the tumor) and micro-metastasis are relevant when considering the risk factors of biochemical recurrence in cases with PSM. This model may support decision making in omitting adjuvant therapies in patients within this low-risk group.

## Supplementary Information

Below is the link to the electronic supplementary material.

Supplementary file1 (PPTX 114 KB)

## Data Availability

No datasets were generated or analysed during the current study.
