# Supplementary material for: Risk factors and risk-stratification model of biochemical failure in patients with positive surgical margin after robot-assisted radical prostatectomy
Source: J Robot Surg. 2025 Jun 4;19(1):261. doi: 10.1007/s11701-025-02426-x (PMC12137452; doi:10.1007/s11701-025-02426-x)
Supplement: Supplementary file 1 — Supplementary file1 (PPTX 114 KB) [file 11701_2025_2426_MOESM1_ESM.pptx]

## Slide 1
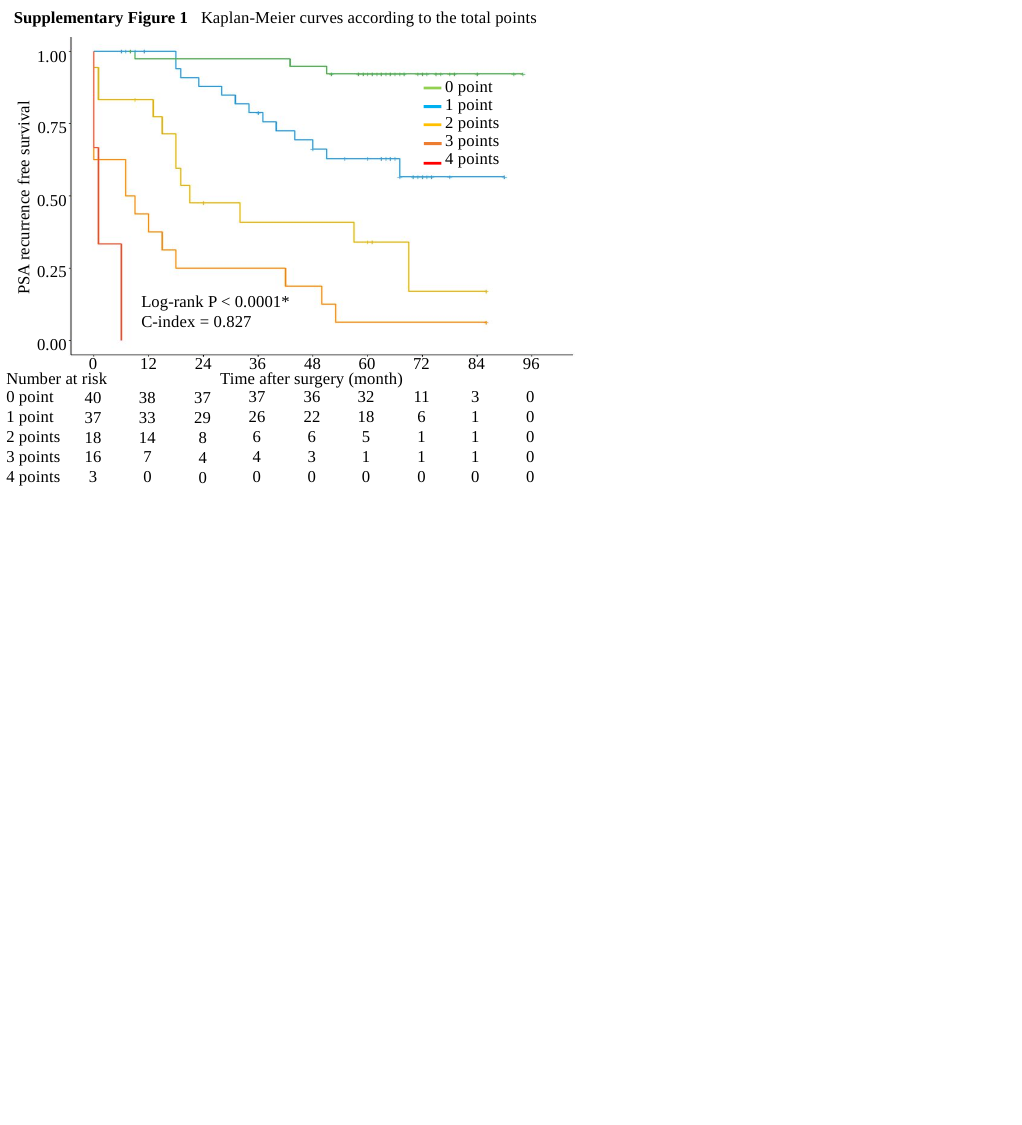

Supplementary Figure 1 Kaplan-Meier curves according to the total points
1.00
0 point
1 point
2 points
3 points
4 points
0.75
0.50
PSA recurrence free survival
0.25
Log-rank P < 0.0001*
C-index = 0.827
0.00
84
0
12
96
24
60
36
48
72
Time after surgery (month)
Number at risk
0
0
0
0
0
0 point
1 point
2 points
3 points
4 points
11
6
1
1
0
3
1
1
1
0
32
18
5
1
0
37
26
6
4
0
36
22
6
3
0
40
37
18
16
3
38
33
14
7
0
37
29
8
4
0

## Slide 2
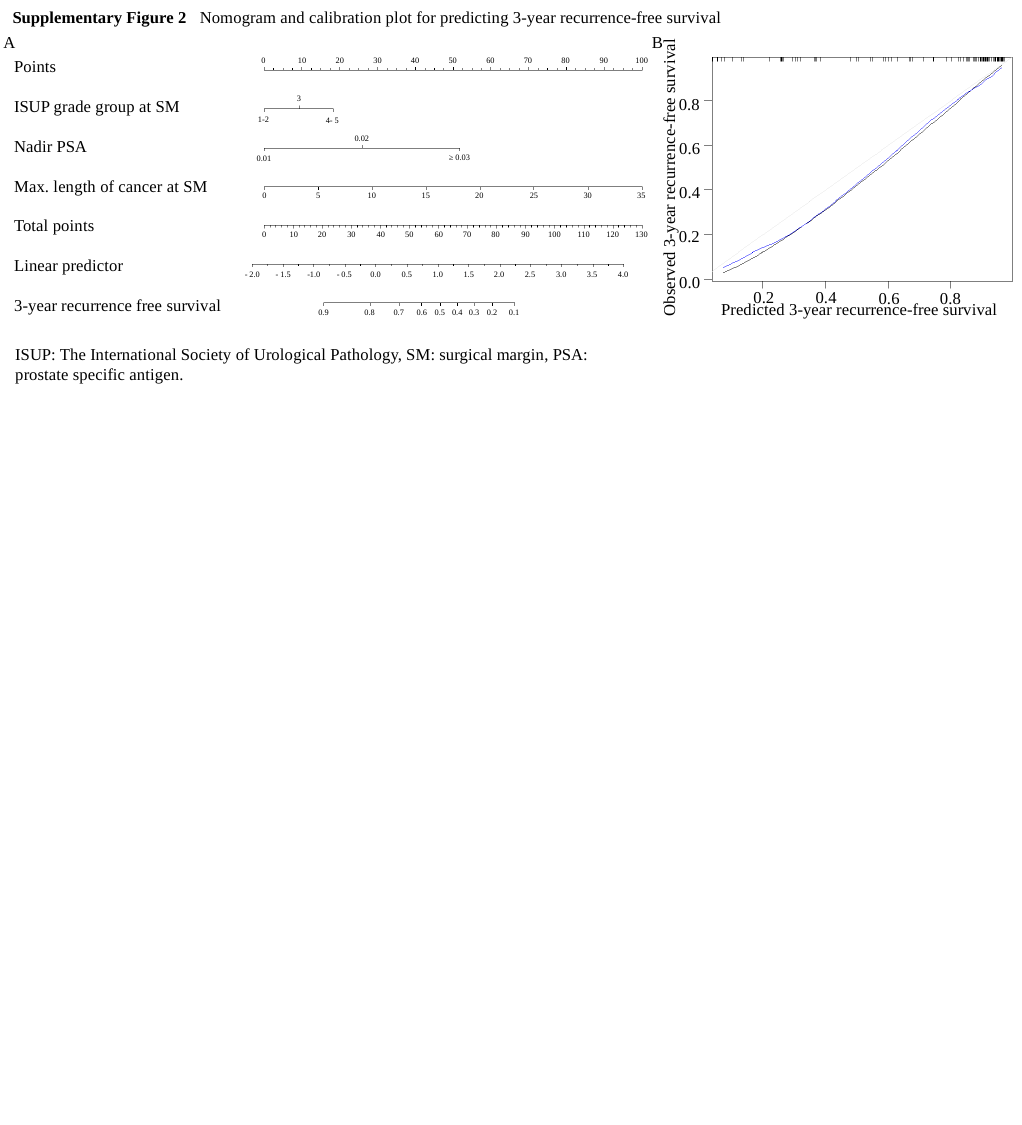

Supplementary Figure 2 Nomogram and calibration plot for predicting 3-year recurrence-free survival
A
B
Points
ISUP grade group at SM
Nadir PSA
Max. length of cancer at SM
Total points
Linear predictor
3-year recurrence free survival
100
80
90
30
40
50
60
70
0
20
10
0.8
3
1-2
4- 5
0.6
0.02
≥ 0.03
0.01
Observed 3-year recurrence-free survival
0.4
30
35
20
25
5
10
15
0
0.2
130
100
110
120
80
90
30
40
50
60
70
0
20
10
0.0
3.5
4.0
2.5
3.0
2.0
1.0
1.5
0.0
0.5
- 1.5
-1.0
- 0.5
- 2.0
0.2
0.4
0.6
0.8
Predicted 3-year recurrence-free survival
0.7
0.6
0.5
0.4
0.3
0.2
0.1
0.9
0.8
ISUP: The International Society of Urological Pathology, SM: surgical margin, PSA: prostate specific antigen.
